# Clinical outcome of therapy‐related acute myeloid leukemia patients. Real‐life experience in a University Hospital and a Cancer Center in France

**DOI:** 10.1002/cam4.6322

**Published:** 2023-08-07

**Authors:** Amine Belhabri, Mael Heiblig, Stephane Morisset, Liliana Vila, Clémence Santana, Emmanuelle Nicolas‐Virelizier, Sandrine Hayette, Isabelle Tigaud, Adriana Plesa, Hélène Labussiere‐Wallet, Mohamad Sobh, Anne‐Sophie Michallet, Balsat Marie, Franck‐Emmanuel Nicolini, Yann Guillermin, Fossard Gaëlle, Laure Lebras, Philippe Rey, Lucie Jauffret‐Bertholon, Marie‐Charlotte Laude, Loron Sandrine, Mauricette Michallet

**Affiliations:** ^1^ Department of Hematology Leon Berard Cancer Center Lyon France; ^2^ Department of Hematology University Hospital Lyon Sud Pierre Benite France; ^3^ Biostatistics Department Leon Berard Cancer Center Lyon France; ^4^ Department of biology – GHS University Hospital Lyon Sud Pierre Benite France; ^5^ Research Advisor, Faculty of Medicine University of Ottawa Ottawa Canada

**Keywords:** clinical outcome, real‐life management, therapy‐related AML

## Abstract

**Background:**

t‐AML occurs after a primary malignancy treatment and retains a poor prognosis.

**Aims:**

To determine the impact of primary malignancies, therapeutic strategies, and prognostic factors on clinical outcomes of t‐AML.

**Results:**

A total of 112 adult patients were included in this study. Fifty‐Five patients received intensive chemotherapy (IC), 33 non‐IC, and 24 best supportive care. At t‐AML diagnosis, 42% and 44% of patients presented an unfavorable karyotype and unfavorable 2010 ELN risk profile, respectively. Among treated patients (*n* = 88), 43 (49%) achieved complete remission: four out of 33 (12%) and 39 out of 55 (71%) in non‐IC and IC groups, respectively. With a median follow‐up of 5.5 months, the median overall survival (OS) and disease‐free survival (DFS) for the whole population were 9 months and 6.3 months, respectively, and for the 88 treated patients 13.5 months and 8.2 months, respectively. Univariate analysis on OS and DFS showed a significant impact of high white blood cells (WBC) and blast counts at diagnosis, unfavorable karyotype and ELN classification. Multivariate analysis showed a negative impact of WBC count at diagnosis and a positive impact of chemotherapy on OS and DFS in the whole population. It also showed a negative impact of previous auto‐HCT and high WBC count on OS and DFS and of IC on OS in treated patients which disappeared when we considered only confounding variables (age, previous cancers, marrow blasts, and 2010 ELN classification). In a pair‐matched analysis comparing IC treated t‐AML with de novo AML, there was no difference of OS and DFS between the two populations.

**Conclusion:**

We showed, in this study that t‐AML patients with unfavorable features represented almost half of the population. Best outcomes obtained in patients receiving IC must be balanced by known confounding variables and should be improved by using new innovative agents and therapeutic strategies.

## Graphical abstract

Therapy‐related AML patients had for the majority of them unfavorable features. Among whole population of our study 50% of patients received intensive chemotherapy (71% of CR), 30% nonintensive chemotherapy (12% of CR) and the rest, best supportive care. Median OS and Median DFS were 9 months and 6.3 months in the whole population and 13.5 months and 8.2 months in treated population. Best outcomes obtained in patients receiving IC must be balanced by known confounding variables and should be improved by using new innovative agents and therapeutic strategies.

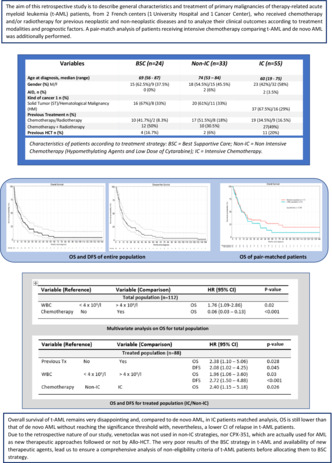

## 1 INTRODUCTION

Therapy‐related myeloid neoplasms (t‐MNs) including therapy‐related acute myeloid leukemias (t‐AML) and therapy‐related myelodysplastic syndromes (t‐MDS) are considered as a distinct category in the 2016 revised World Health Organization (WHO) classification.^1^ This entity is characterized by myeloid neoplasms emerging after treatment for a primary malignancy and associated with a poor prognosis.^2^, ^3^ Among newly diagnosed AML and MDS cases, 10% and 20% are therapy‐related and are generally considered as an independent adverse prognostic factor.^2^, ^3^, ^4^, ^5^ Therapy‐related AML have been thought to be the direct consequence of mutational events induced by cytotoxic therapy and/or targeted therapy, probably promoted by pre‐existing conditions such as clonal hematopoiesis.^6^, ^7^, ^8^ The most common subtype occurs 5–7 years after first exposure to alkylating agents or radiation, is often preceded by MDS, and frequently accompanied by chromosomes 5 and/or 7 abnormalities, complex karyotype, and TP53 mutations.^9^, ^10^ Otherwise, t‐AML after topoisomerase II inhibitors occurs 1–3 years after first exposure, rarely preceded by MDS and usually associated with multiple gene rearrangements.^11^, ^12^ Results comparing t‐AML/MDS and de novo AML/MDS, have been conflicting so far.^13^ Because of similarity regarding cytogenetic features of de novo MN and t‐MN, patients have been treated for a long time in the same way.^14^ Nowadays, according to knowledge of molecular markers and innovative therapies, new strategies were proposed regarding de novo and t‐MN.

Because over time, the incidence of t‐MN is increasing as more patients survive their primary cancers, a better description of the biologic and prognostic factors associated with this entity is highly warranted.^15^, ^16^ Survival of patients with t‐AML remains poor and some retrospective studies have identified many factors that contribute to poor clinical outcomes.^17^, ^18^ In this subset of patients, there is no clear guidelines regarding frontline treatment strategies which are usually based on IC^13^ or non‐IC as hypomethylating agents (HMA) associated to Bcl2 inhibitors^19^ and allogeneic hematopoietic cell transplantation (Allo‐HCT).^20^, ^21^ Therapy‐related AML patients have often been excluded from frontline clinical trials until availability of liposomal daunorubicin and cytarabine association (Vyxeos®) which demonstrated encouraging results for this subset of patients.^22^

The aim of this retrospective study is to describe general characteristics and treatment modalities of primary malignancies of 112 consecutive t‐AML patients, from two French centers (one University Hospital and one Cancer Center), who received chemotherapy and/or radiotherapy for previous neoplastic and non‐neoplastic diseases and to analyze their clinical outcomes according to treatment modalities and prognostic factors.

## 2 PATIENTS AND METHODS

### 2.1 General and biological characteristics of patients

Between 01/01/2006 and 01/06/2019, we analyzed retrospectively, in two French centers, 112 adult patients diagnosed with t‐AML according to 2016 revised WHO criteria^1^ and occurring after treatment of solid tumors (ST), hematologic malignancies (HM), or autoimmune diseases (AID). At leukemia diagnosis, we performed peripheral blood count and bone marrow morphology with blast percentage. Samples of bone marrow and blood were also examined for cytogenetic abnormalities. Cytogenetic and molecular risk profiles were recorded according to 2010 European Leukemia Net (ELN) classification.^23^ Next generation sequencing (NGS)^24^ was not available in our two centers until 2016 and was done subsequently for only 13 patients which explained that 2017 and 2022 ELN risk stratifications^25^, ^26^ by genetics were not used in our study. Mixed‐lineage leukemia (MLL) gene fusion and MECOM1 positivity were detected by fluorescence in situ hybridization (FISH). Since 2006, available leukemia samples were analyzed for mutations in the FLT3 protein domains (TKD and ITD), NPM1, IDH1/2, and Evi1 overexpression, using real‐time quantitative reverse transcriptase‐polymerase chain reaction (RT‐PCR).^27^

### 2.2 Treatment of previous malignancies and t‐AML

Patients received chemotherapy and/or radiotherapy as treatment for their primary malignancy and for some of them, high dose chemotherapy followed by autologous hematopoietic cell transplantation (Auto‐HCT). All patients eligible for IC were treated by an anthracycline and cytarabine‐based induction chemotherapy regimen (*N* = 55 including CPX‐351 in seven patients). Patients achieving complete remission (CR) after one or two courses of induction received consolidation chemotherapy. Patients unfit for intensive chemotherapy received frontline non‐IC: nine low dose cytarabine (LDC) and 24 hypomethylating agents (HMA). All HMA patients received azacytidine. Policy regarding transfusions, antibiotics, and antifungal prophylaxis were determined according to established local practice. Twenty‐four patients received best supportive care (BSC) with for some patient's administration of hydroxyurea or 6‐mercaptopurine.

### 2.3 Therapy‐related AML response criteria

CR was defined according to 2017 ELN criteria.^25^ Patients in partial response or failure after treatment were considered as nonresponders. Measurable residual disease (MRD) response by flow cytometry (FCM) or RT‐qPCR, when possible, has not been included in this study.

### 2.4 Statistical analysis

We performed a global descriptive analysis of the baseline characteristics of the cohort and a specific analysis of each treatment group. Continuous variables were summarized as median (min; max) and [interquartile range] and compared between groups with a Kruskal–Wallis test. Qualitative variables were synthetized as counts and percentages and compared between groups by a Pearson's chi‐square test (with Monte Carlo simulations if one count is below 5).

Overall survival (OS) was defined as the time from diagnosis to death at last follow‐up and disease‐free survival (DFS) as the time since diagnosis to death or relapse date. Patients with no events or allografted before whichever event were censored. Survival curves were illustrated with the Kaplan–Meier method and compared with a log‐rank test. Univariate and multivariate analyses on survival were done using Cox regression. Relapse was illustrated thanks to the cumulative incidence (CI) function. Univariate and multivariate analyses on CI of relapse was done using the Fine and Gray regression.

The bilateral level of significance was set at 5%. All the statistical analyses and graphics were made under the R program (v3.5.1) and its “survival,” “cmprsk,” and “ggplot2” packages.

A pair‐matched control analysis of t‐AML patients treated by IC with de novo AML also treated by IC was matched for age, cytogenetic features, and 2010 ELN classification.

## 3 RESULTS

### 3.1 Patient characteristics

Among 112 patients included in the study, 56 were males (50%) with a median age of 68 years (19–87) at diagnosis of t‐AML. Before t‐AML, 108 patients presented a first cancer: 73 patients ST (64.5%), 35 HM (30.5%) from whom six patients (5.4%) developed a second cancer (four in ST and two in HM). Four patients presented AID (3.6%). Forty‐six patients (41%) received chemotherapy for their primary disease (alkylating agents, topoisomerase II inhibitors alone or in combination or other agents), 17 (15%) radiotherapy and 49 (44%) radiochemotherapy. Seventeen patients (15%) underwent Auto‐HCT after high dose chemotherapy (two ST and 15 HM). All t‐AML after AID were treated by long‐term methotrexate. Median interval from treatment of previous ST, HM, and AID to t‐AML diagnosis was 5.3 years (0.4–34.3) (Table 1).

**TABLE 1:** Descriptive analysis of total population according to therapeutic strategy.

Variables	BSC (n = 24)	Non‐IC (n = 33)	IC (n = 55)	Total (n = 112)
Age at diagnosis, median (range)	69 (56–87)	74 (53–84)	60 (19–75)	68 (19–87)^a^
Gender (%) M/F	15 (62.5%)/9 (37.5%)	18 (54.5%)/15 (45.5%)	23 (42%)/32 (58%)	56 (50%)/56 (50%)
Kind of cancer 1, n (%)				
Breast/Ovarian	6 (25%)/0 (0%)	8 (24%)/2 (6%)	19 (35%)/1 (2%)	33 (30%)/3 (2.8%)
Gut/Uro/ORL	5(20.8%)/1 (4.2%)/0 (0%)	1 (3%)/2 (6%)/5 (15%)	3 (5.5%)/4 (7%)/1 (2%)	9 (8%)/7 (6.3%)/6 (5%)
Non Hodgkin Lymphoma/Hodgkin Lymphoma/Myeloma/CLL/APL	4(16.7%)/1 (4.2%)/2 (8.3%)/1 (4.2%)/0(0%)	9 (27%)/1 (3%)/0 (0%)/1 (3%)/0 (0%)	6 (11%)/4 (7%)/3 (5.5%)/2 (3.5%)/1(1.5%)	19 (17%)/6 (5%)/5 (4.5%)/4 (3.6%)/1(0.5%)
Sarcoma/Lung/Melanoma	4 (16.7%)/0 (0%)/0(0%)	1 (3%)/1 (3%)/0 (0%)	6 (11%)/2 (3.5%)/1 (1.5%)	11 (10%)/3 (2.8%)
AID, n (%)	0 (0%)	2 (6%)	2 (3.5%)	4 (3.5%)
Kind of cancer 1 n (%)				
Solid Tumor (ST)/Hematological Malignancy (HM)	16 (67%)/8 (33%)	20 (61%)/11 (33%)	37 (67.5%)/16 (29%)	73 (65.5%)/35 (31%)
Number of cancer, n (%) 1/2	22 (91.7%)/2 (8.3%)	29 (88%)/4 (12%)	54 (98%)/1 (2%)	105 (94%)/7 (6%)
Kind of cancer 2, n (%)	2	3	1	6
Ovarian/Uro/Gut	1 (50%)/1 (50%)/0 (0%)	1 (33%)/0 (0%)/1 (33%)	‐	2 (28%)/1 (14%)/1 (14%)
Non Hodgkin Lymphoma/Myeloma	–	1 (33%)/0 (0%)	0 (0%)/1 (100%)	1 (14%)/1 (14%)
Previous Treatment n (%)				
Chemotherapy/Radiotherapy	10 (41.7%)/2 (8.3%)	17 (51.5%)/8 (18%)	19 (34.5%)/9 (16.5%)	46 (41%)/19 (17%)
Chemotherapy + Radiotherapy	12 (50%)	10 (30.5%)	27(49%)	49 (44%)
Previous chemotherapy, n	20	19	42	81
Alkylator agents	1 (5%)	5 (26.5%)	6 (14%)	12 (15%)
Topoisomerase inhibitors	4 (20%)	1 (5%)	2 (5%)	7 (9%)
Alkylator agents + Topoisomerase inhibitors	15 (75%)	13 (68.5%)	34 (81%)	62 (76%)
Previous HCT n (%)	4 (16.7%)	2 (6%)	11 (20%)	17 (15%)
Interval cancer1/AML(y), median (range) [1st–3rd Q] (n = 45)	6.34 (0.43; 20.85) [4.59–12.21]	4.43 (1.93; 23.14) [3.75–11.19]	4.79 (1.14; 34.28) [1.85–6.82]	5.34 (0.43; 34.28) [2.85–10.7]
WBC (10^9^/l), median (range)) [1st–3rd Q] (n = 74)	4.5 (1; 400) [1.7–35.7]	2.8 (1.1; 188) [1.8–6.7]	4 (0.6; 313) [2.04–17.96]	3.7 (0.6; 400) [1.8–15.8]
% blasts blood, median (range) [1st–3rd Q] (n = 72)	10 (0; 95) [5–66]	8 (0; 93) [3–41]	15 (0; 97) [2–51]	10 (0; 97) [2–55]
% blasts BM, median (range) [1st–3rd Q] (n = 81)	60 (10; 95) [36–87]	45 (20; 95) [30–61]	60 (12; 100) [35–80]	50 (10; 100) [33–80]
Hemoglobin (g/dl), median (range) [1st–3rd Q] (n = 73)	8.9 (4.9; 12.3) [8.3–10.1]	9.1 (6.4; 12.7) [8.3–10.2]	9.9 (5.4; 15) [9.2–10.9]	9.5 (4.9; 15) [8.4–10.6]^b^
Platelets (10^9^/l), median (range) [1st–3rd Q] (n = 74)	40 (13; 217) [30–58]	56 (10; 140) [29–88]	58 (8; 739) [32–108]	54 (8; 739) [30–94]
Karyotype, n (%)	18 (75%)	31 (94%)	55 (100%)	104 (93%)
Favorable /Intermediate/Unfavorable/Failure	0 (0%)/6 (33.5%)/11 (61%)/1 (5.5%)	1 (3%)/9 (29%)/15 (48.5%)/6(19.5%)	3 (5.5%)/31 (56%)/18 (33%)/3 (5.5%)	4 (4%)/46 (44.5%)/44 (42%)/10 (9.5%)^b^
Molecular biology (RT‐PCR), n (%)	13 (54%)	28 (85%)	51 (93%)	92 (82%)
FLT3/NPM1/EVI1/IDH1 + IDH2	2 (15.5%)/1 (8%)/ 1 (8%)/0 (%)	3 (11%)/3 (11%)/ 5 (18%)/0 (0%)	4(8%)/6 (12%)/16 (31.5%)/1 (2%)	9 (10%)/10 (11%)/22 (24%)/1 (1%)
2010 ELN, n (%)	11 (46%)	21 (64%)	48 (87%)	80 (71.5%)^a^
Favorable	0 (0%)	3 (14%)	6 (12.5%)	9 (11%)
Intermediate‐1/Intermediate‐2	1 (9%)/5 (46%)	3 (14%)/3 (14%)	11 (23%)/13 (27%)	15 (19%)/21 (26%)
Unfavorable	5 (46%)	12 (57%)	18 (37.5%)	35 (44%)
Complete Remission (CR), n (%)	0 (0%)	4 (12%)	39 (71%)	43 (38%)^a^
Allo HCT after induction, n (%)	0 (0%)	1 (3%)	14 (25.5%)	15 (13.5%)^a^
Interval AML‐HCT (months), median (range) [1st–3rd Q]	54 (3.8; 264) [26–140]	66 (9; 678) [34–106]	46 (8.5; 243.5) [33–79]	51 (3.8; 678) [32.5–95]
Interval AML‐Last FU (months), median (range) [1st–3rd Q]	1 (0; 5) [0.7–1.4]	7 (0.3; 40.5) [5–17]	12.5 (0.7; 144.5) [5–34.5]	6.6 (0; 144) [2–19]

Abbreviations: AID, autoimmune disease; APL, acute promyelocytic leukemia; BSC, best supportive care; Non‐IC, nonintensive chemotherapy (Hypomethylating agents and low dose cytarabine); IC, intensive chemotherapy; Allo HCT: allogeneic hematopoietic cell transplantation; WBC, white blood count; RT‐PCR, reverse transcriptase‐polymerase chain reaction; FU, follow‐up; Q, Quartile.

^a^ p < 0.001.

^b^ p = 0.045.

*Note*: When we considered only treated population comparing IC versus non‐IC: *p* < 0.001 (age, gender, and Allo‐HCT) *p* = 0.038 (karyotype and 2010 ELN).

Therapy‐related AML after AID had similar characteristics to ST and HM populations in terms of delay of t‐AML occurrence, demographic and genetics features, response to therapy and outcome. At t‐AML diagnosis, cytogenetics was unfavorable in 44 patients (42%) with 28 (64%) complex karyotypes and 16 no complex karyotypes (three patients with 5q deletion, five with monosomy 7, three with 7q deletion, two with both chromosome abnormalities, and three patients with other abnormalities). Nine patients presented chromosome 17 abnormalities (two deletions and seven monosomies) and all belonged to complex karyotypes. Considering t‐AML karyotype, among intermediate risk patients, 70% were observed after ST, 17% after HM, and 13% after AID. In unfavorable risk patients, 59% were observed after ST and 41% after HM. (*p* = 0.03).

Regarding molecular alterations, *FLT3‐ITD*, *FLT3‐TKD*, *NPM1*, and *IDH* mutations were observed in 6.9%, 2.3%, 11.5%, and 2.3% of patients, respectively. *MECOM1* was overexpressed in 25% (22/88) of cases. *MECOM1* overexpression was associated with 11q23 and chromosome 3 rearrangements in 9/22 (40.9%) and 3/22 (13.6%) patients, respectively. According to 2010 ELN classification, 11%, 45%, and 44% of patients were favorable, intermediate (1 and 2), and unfavorable, respectively. NGS was available for only 13 patients. Median number of mutations was 2 (1–5). Patients with complex karyotype had lower mutation burden (median: 1.9 [1–3]) than patients with noncomplex karyotype (median: 3.2 [1–5]; *p* = 0.17) (Figure S1A). *TP53* mutation was highly enriched (6/13) in this t‐AML population including three patients carrying chromosome 17 abnormalities (one patient with complex karyotype and two patients with intermediate karyotype). Co‐occurring mutations were summarized in Figure S1B. In *TP53* mutated patients, there was a strong co‐occurrence with RTK pathway mutations, especially RAS pathway (Figure S1A). Overall, 55 (49%) and 33 (29.5%) received IC and non‐IC [24 (21.5%) HMA and 9 (8%) LDC], respectively. Twenty‐four patients (21.5%) were not treated and therefore received BSC. Among treated patients (*n* = 88) (IC + non‐IC), 43 patients (49%) achieved CR: four out of 33 (12%) in non‐IC patients (three in HMA and one in LDC) and 39 out of 55 (71%) in IC patients. Fifteen patients (17%) underwent Allo‐HCT with a median interval between AML diagnosis and Allo‐HCT of 4.7 months (2.6–17.5) (Table 1).

When we considered treated patients IC + Non‐IC (*n* = 88), we observed differences concerning age (*p* < 0.001), 2010 ELN classification with more favorable, intermediate‐1 and intermediate‐2 and less unfavorable in IC group (*p* = 0.04), number of previous cancer (*p* = 0.06), and bone marrow blast percentage (*p* = 0.08) (Table 2).

**TABLE 2:** Univariate analysis on OS and DFS for total population (*n* = 112 patients).

Variable	Available data	Reference	Modality	OS		DFS	
				HR [95% CI]	Cox *p* value	HR [95% CI]	Cox *p* value
Age at diagnosis	100%	Continuous variable		1.01 [0.99–1.03]	0.199	1.01 [0.99–1.02]	0.185
Gender	100%	M	F	0.68 [0.44–1.06]	0.090	0.80 [0.53–1.20]	0.289
Kind of Cancer 1	99.11%	Breast Cancer	Ovarian	0.37 [0.09–1.60]	0.184	0.33 [0.08–1.42]	0.136
			Gut	1.21 [0.53–2.76]	0.645	1.37 [0.63–2.97]	0.421
			Uro/Prostate	0.74 [0.28–2.01]	0.560	0.63 [0.24–1.68]	0.358
			Hodgkin Disease	0.84 [0.29–2.47]	0.758	0.63 [0.24–1.67]	0.353
			Myeloma	1.80 [0.61–5.28]	0.288	1.43 [0.49–4.16]	0.509
			NHLymphoma	0.71 [0.36–1.39]	0.313	0.70 [0.37–1.31]	0.259
			Sarcoma	1.81 [0.83–3.96]	0.136	1.08 [0.52–2.27]	0.834
			AID+APL + Melanoma	0.24 [0.06–1.02]	0.053	0.28 [0.08–0.95]	0.041
			ORL	1.14 [0.46–2.84]	0.775	0.87 [0.35–2.13]	0.754
			CLL	1.67 [0.49–5.62]	0.410	1.81 [0.62–5.28]	0.274
			Lung	1.67 [0.39–7.18]	0.491	0.68 [0.20–2.31]	0.540
Previous Chemotherapy	100%	No	Yes	1.14 [0.64–2.03]	0.657	1.27 [0.72–2.25]	0.405
Previous Transplant	100%	No	Yes	1.68 [0.95–2.96]	0.074	1.54 [0.89–2.66]	0.120
Previous Radiotherapy	100%	No	Yes	1.19 [0.76–1.87]	0.455	1.06 [0.71–1.60]	0.769
Interval cancer1 – AML (y)	54.46%	Continuous variable		1.00 [0.97–1.04]	0.932	1.00 [0.96–1.03]	0.942
WBC	85.71%	Continuous variable		1.00 [1.00–1.01]	0.009	1.00 [1.00–1.01]	0.006
% blastes in Blood	83.04%	Continuous variable		1.01 [1.00–1.01]	0.042	1.01 [1.00–1.02]	0.001
% blastes in BM	91.96%	Continuous variable		1.00 [0.99–1.01]	0.759	1.01 [1.00–1.01]	0.184
Treatment of AML	100%	No	Yes	0.05 [0.03–0.11]	<0.001	–	–
Karyotype	92.86%	Favorable—Intermediate	Unfavorable	1.94 [1.17–3.22]	0.010	1.65 [1.04–2.60]	0.032
FLT3 (ITD‐TKD)	81.25%	No	Yes	1.24 [0.61–2.53]	0.555	1.28 [0.63–2.57]	0.497
NPM1	82.14%	No	Yes	0.71 [0.30–1.65]	0.420	0.71 [0.33–1.56]	0.398
Evi1	73.21%	No	Yes	0.73 [0.40–1.35]	0.320	0.75 [0.43–1.30]	0.302
ELN 2010	91.96%	Favorable—Intermediate‐1	Intermediate‐2	2.24 [1.09–4.63]	0.029	1.76 [0.91–3.40]	0.095
			Unfavorable	1.99 [1.02–3.90]	0.044	1.61 [0.90–2.91]	0.111

### 3.2 Clinical outcome

**FIGURE 1:**
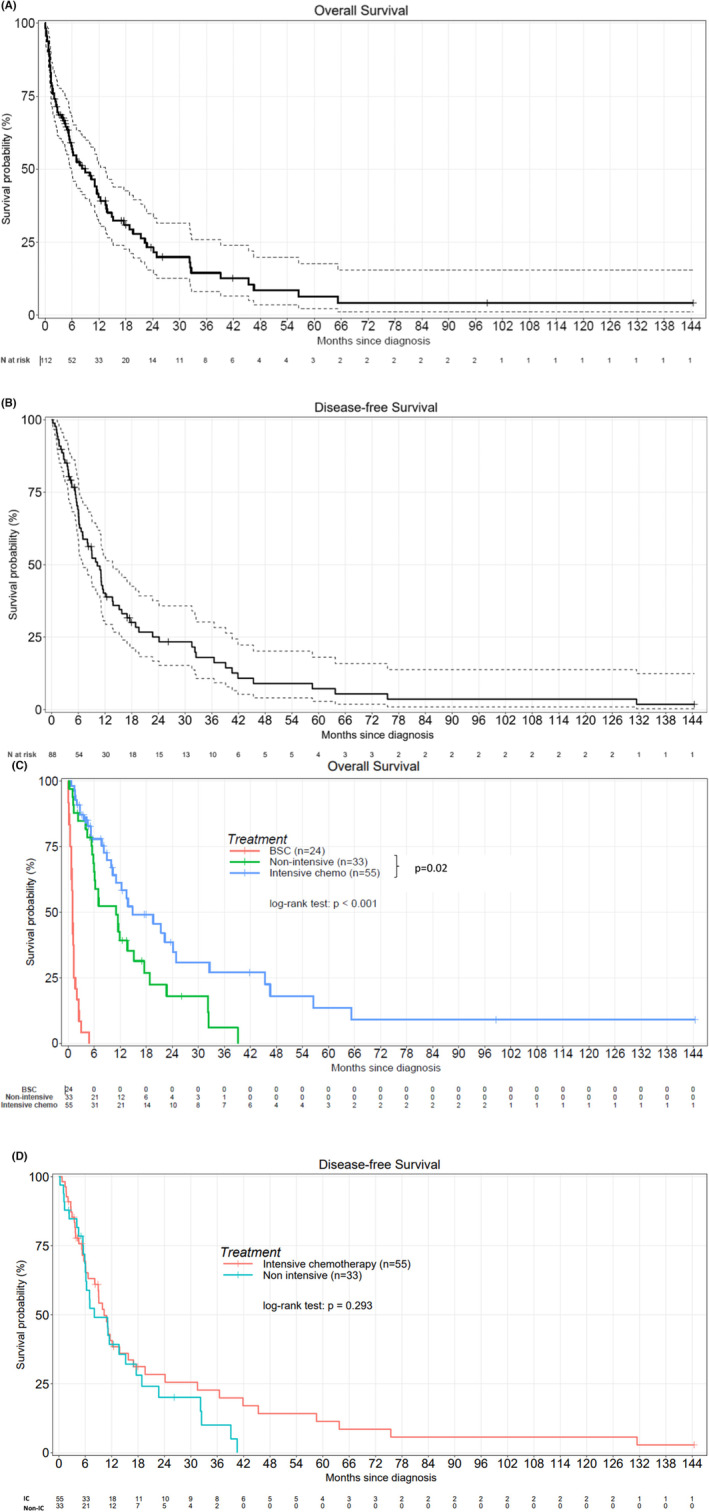
Kaplan Meier curves evaluatiing OS and DFS (A) OS of whole population, (B) DFS of treated population, (C) OS of whole population according to treatment groups, (D) DFS of treated population according to treatment intensity.

**FIGURE 2:**
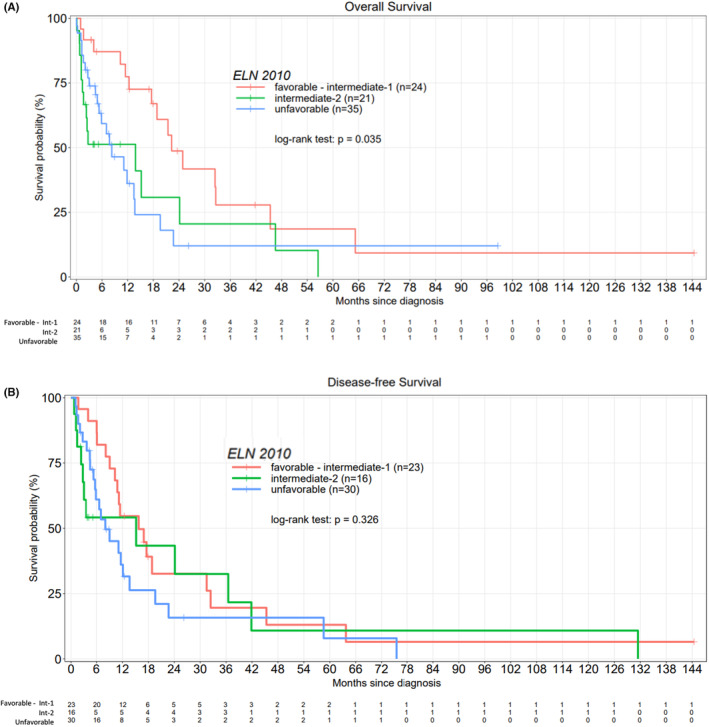
Kaplan‐Meier curves evaluatiing OS and DFS according to 2010 ELN classification (A) OS of whole population, (B) DFS of treated population.

With a median follow‐up of 5.5 months (0–144), the median OS for the whole population and DFS for the treated population were 9 months (5.9–13.5) and 6.3 months (5.3–10.3), respectively (Figure 1A,B). There was a significant difference between the three groups (entire population: BSC vs. non‐IC vs. IC) in terms of OS (*p* < 0.001) as well as between IC (median of 14.8 months) and non‐IC (median of 11 months) groups (*p* = 0.02) (Figure 1C). There was no difference between IC (median of 10.3 months) and non‐IC (median of 8 months) groups in terms of DFS (Figure 1D). Univariate analysis showed a significant impact of the act of receiving chemotherapy, high WBC and high blast counts, unfavorable karyotype, and 2010 ELN (Table 3). Overall survival (*p* = 0.035) and DFS (*p* = 0.326) according to 2010 ELN classification were shown in Figure 2B.

**TABLE 3:** Multivariate analysis on OS (A) for total population (*n* = 112 patients) and OS and DFS (B) for treated population (IC/Non‐IC) (*n* = 88 patients).

Variable (Reference)		Variable (Comparison)		HR (95% CI)	*p* value
(A) Total population (n = 112)					
WBC	<4 x 10^9^/l	>4 x 10^9^/l	OS	1.76 (1.09–2.86)	0.02
Chemotherapy	No	Yes	OS	0.06 (0.03–0.13)	<0.001
(B) Treated population (n = 88)					
Previous Tx	No	Yes	OS DFS	2.38 (1.10–5.06) 2.08 (1.02–4.25)	0.028 0.045
WBC	<4 x 10^9^/l	> 4 x 10^9^/l	OS DFS	1.96 (1.06–3.60) 2.72 (1.50–4.88)	0.03 <0.001
Chemotherapy	Non‐IC	IC	OS	2.40 (1.15–5.18)	0.026

**FIGURE 3:**
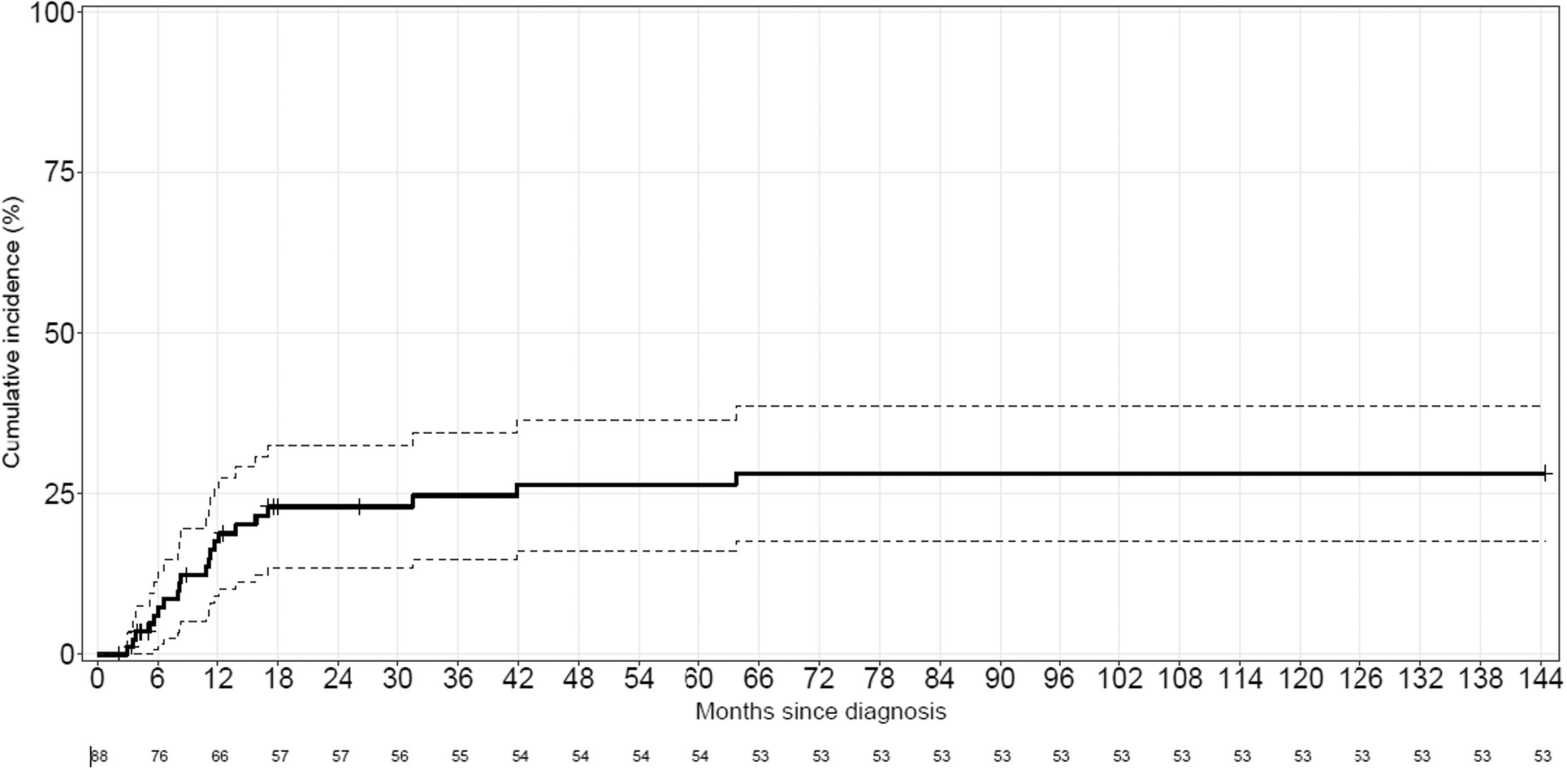
Cumulative incidence of relapse in treated population.

In the 88 treated patients with either IC or non‐IC, 43 achieved CR [39 out of 55 (71%) and four out of 33 (12%) in IC and non‐IC group, respectively]. Among these 43 patients, 13 are alive at the last follow‐up (nine in CR and four in relapse) and 30 died [16 from relapse (53%) and 14 from nonrelapse causes]. Among the remaining 45 patients who did not achieve CR, 41 patients (91%) died from disease and four patients were lost of follow‐up. In the treated population, with a median follow‐up of 8.2 months (0.3–144), the median OS and DFS were 13.5 months (10.3–19.6) and 8.2 months (7–13.7), respectively. When we considered only patients receiving IC, the median OS was 14.8 months (11–32.6) and median DFS was 9.9 months (6.6–15.8). The cumulative incidence (CI) of relapse in treated population was 17.5% (9–26), 24.7% (14.8–34.5), and 26.4% (16–36.6) at 12, 36, and 60 months, respectively (Figure 3).

In multivariate analysis of the entire cohort (Table 3A), we observed a significant negative impact of WBC count >4 Giga/L on OS (*p* = 0.02) and a significant positive impact of the act of receiving chemotherapy on OS (*p* < 0.001).

In multivariate analysis including only treated patients (Table 3B), previous Auto‐HCT had a significant negative impact on OS (*p* = 0.028) and DFS (*p* = 0.045), WBC count >4 G/L had a significant negative impact on OS (*p* = 0.03) and DFS (*p* < 0.001) and type of chemotherapy (IC vs. non‐IC) had a significant negative impact on OS (*p* = 0.026). In order to have an unbiased estimate of the effect of chemotherapy intensity, we performed univariate and multivariate analyses on OS concerning only confounding variables (age, number of previous cancers, marrow blast count, and 2010 ELN classification). We observed a persistent significant impact of chemotherapy intensity in univariate which disappeared in multivariate analysis (Table S1).

### 3.3 Results of pair‐matched analysis

**FIGURE 4:**
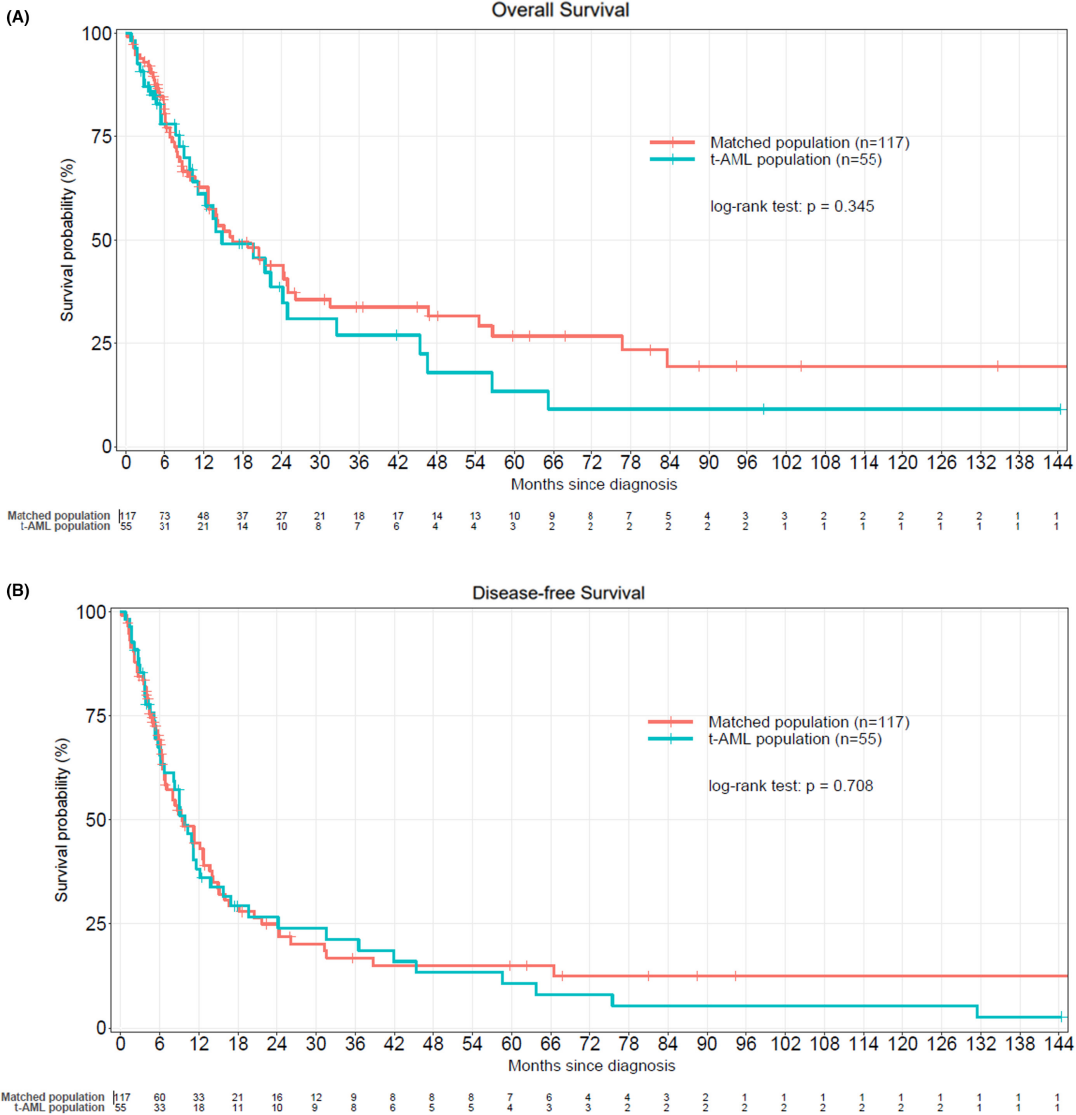
Kaplan‐Meier curves evaluating OS and DFS of pair‐matched populations (IC treated AML vs de novo AML), (A) OS of t‐AML and matched de novo AML, (B) DFS of t‐AML and matched de novo AML.

**FIGURE 5:**
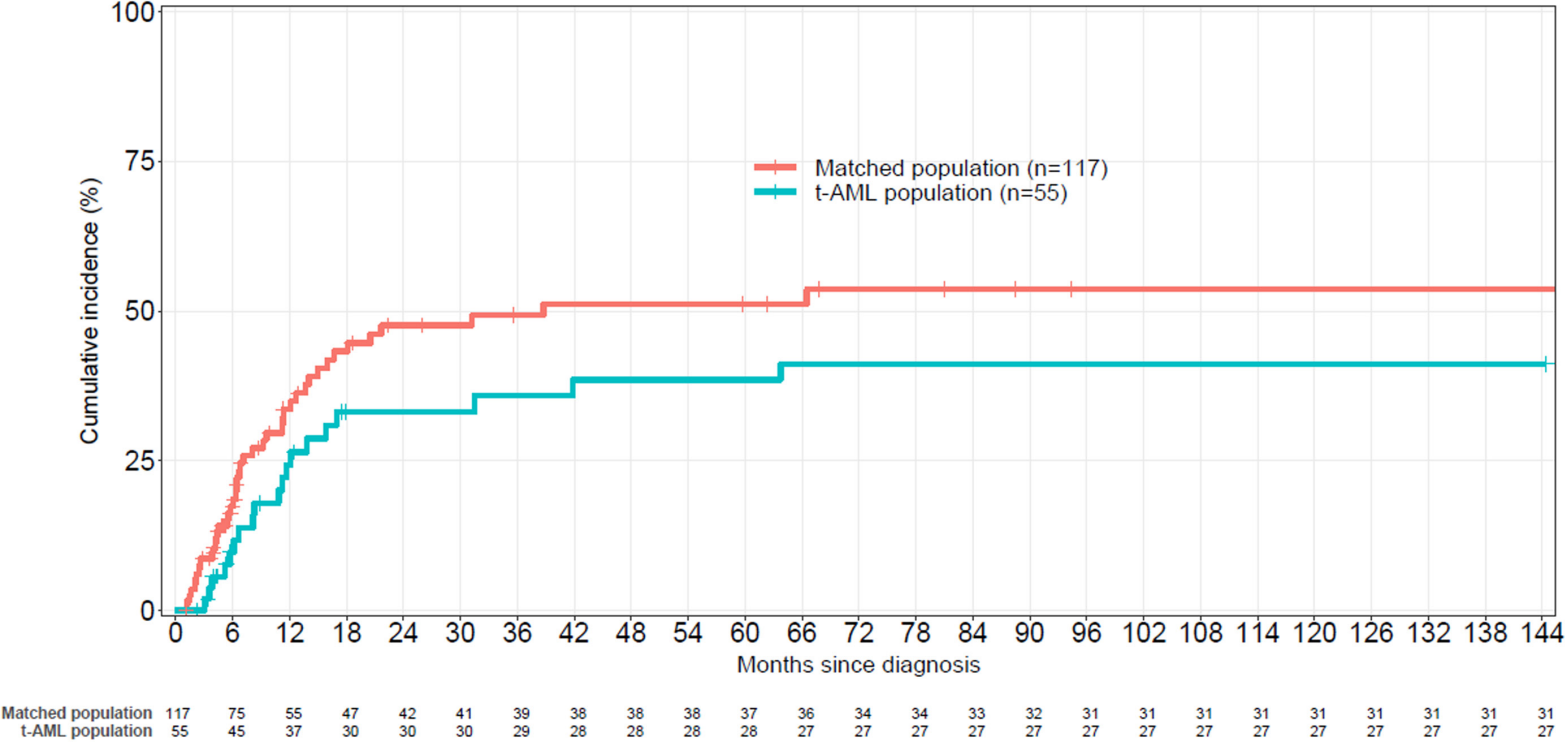
Cumulative incidence of relapse of pair‐matched populations.

In order to compare clinical outcome of de novo AML (*n* = 117) and t‐AML (*n* = 55) treated by IC, we performed a pair‐matched analysis. Characteristics of the two matched cohorts were not significantly different although a difference of age between the two cohorts was observed but which did not reach a significant level (Table S2). Median OS was 16.5 months (12.7–24.9) and 14.8 months (11–32.6) in de novo and t‐AML, respectively (*p* = 0.345). Median DFS was 9.45 months (6.8–13.7) and 9.9 months (6.6–15.8) for de novo and t‐AML, respectively (*p* = 0.708) (Figure 4,B). In addition, there was no difference concerning 2010 ELN classification between t‐AML and *de novo* AML when we analyzed the different prognostic groups (Figure S2). CI of relapse were 47.6% (36.7–58) and 33% (19.5–46.8) at 2 years and 51% (40–62.3) and 38.5% (24–53) at 5 years for de novo and t‐AML patients, respectively, with a lower CI of relapse for t‐AML (Figure 5).

### 3.4 Allogeneic HCT results

According to international recommendations for HCT in AML patients,^28^ 15 patients [five males and 10 females with a median age of 53 years (21–65)] underwent Allo‐HCT in first CR. Before transplantation, 14 patients have received IC, one patient HMA and no patient received CPX‐351. As conditioning regimen, patients received either myeloablative (MAC) (*n* = 2) or reduced intensity conditioning (RIC) (*n* = 13) including antithymocyte globulins (ATG) in nine patients. Hematopoietic cell sources were two bone marrow, one cord blood unit, and 11 peripheral hematopoietic cells, from six geno‐identical donors, four unrelated HLA matched donors, and four unrelated HLA mismatched donors. After transplantation, two patients presented a graft failure and among 13 evaluable patients, 12 achieved a full donor chimerism and one a mixed chimerism. Six out of 13 patients developed acute graft‐versus‐host disease (GVHD) (one grade I, three grade II, and two grade III) and six developed a chronic GVHD (one extensive and five limited). The best response after transplantation was CR in 10 patients. At the last follow‐up, four patients are alive (three in CR and one in relapse) and 11 died (five from relapse, five from transplant related mortality causes, and one from another subsequent malignancy).

## 4 DISCUSSION

In our study and as described in several studies,^17^, ^18^, ^29^ 50%–60% of patients had a ST as primary malignancy with a majority of breast cancer and 30%–40% HM with a majority of lymphoproliferative diseases before occurrence of t‐AML. All patients, as in our analyzed population, received chemotherapy combinations with alkylating agents, topoisomerase II inhibitors, platinum compounds, and/or radiotherapy with variable median interval between primary malignancy and t‐AML^15^, ^17^, ^29^, ^30^ Recent study from AML Swedish registry have showed, among patients treated for ST, lymphoma, and multiple myeloma (MM), 4.5% yearly overtime increase of incidence of t‐AML.^31^ Therapy‐related MN after HM other than lymphoma and MM treated by chemotherapy followed or not by auto‐HCT were less studied.^32^, ^33^, ^34^, ^35^, ^36^ Singh et al have evaluated risk of t‐MN in a very large series of ST and MM patients during three periods of time (from 2000 to 2016), except in melanoma, authors observed in the most recent period, an increase rate of t‐MDS and decrease rate of t‐AML probably due to introduction of nonchemotherapy innovative agents.^37^ A study of t‐MN after radiotherapy alone done by Patel et al has shown that the most common cytogenetic abnormality was a clonal abnormality in chromosome 5 and/or 7 with a worse outcome compared with other cytogenetic groups.^38^ Concerning t‐MN after innovative agents, patients treated for ST, with different peptide receptor radionuclide therapy (PRRT)^39^ and more recently, with Poly (ADP‐ribose) polymerase (PARP) inhibitors, developed also high risk t‐MN.^7^, ^40^ Several studies highlighted the role of genetic mutations which belong to CHIP, found at ST diagnosis, growing within malignancy history and exposure to risk factors with a proven impact on t‐MN development.^6^, ^7^, ^8^, ^10^, ^41^, ^42^, ^43^, ^44^ Concerning general characteristics and genetic features of t‐AML patients, as others, we reported more frequent abnormal karyotypes compared to de novo AML^2^, ^15^, ^17^, ^18^, ^45^, ^46^ and no difference in the incidence and distribution of mutated NPM1 and FLT3‐ITD between t‐AML and de novo AML.^47^ TP53 mutations or 17p abnormalities are frequently observed in t‐MNs and associated with complex karyotype, a reduced probability of achieving response after induction chemotherapy and a poor outcome, as shown by Lindsley et al.^48^ Nowadays, prognosis of TP53 mutated t‐AML, according to new AML classifications^26^ must consider variant allelic frequency, single or multihit characteristic and presence of comutations.^49^ Due to NGS nonavailability at the beginning of our study, we reported only few results of genomic assessment in 13 patients with 2 or more mutations and TP53 mutations in 46% co‐occurring, in half of them with chromosome 17 abnormalities. Concerning treatment of t‐AML, a study about 931 older patients with secondary AML using IC‐based regimens compared to epigenetic and low dose cytarabine regimens did not demonstrate a statistically significant difference in OS except for patients who underwent Allo‐HCT.^50^ In our study, we observed positive impact of treatment with a better median OS than in Chen and Chicago group reports.^15^, ^45^ Nowadays, patients with t‐AML and AML‐MRC could receive CPX‐351 which allowed promising results^22^ and specially for patients who underwent Allo‐HCT. In our study, patients receiving IC had a significant higher OS and DFS in univariate but not in multivariate analysis, compared to those treated by non‐IC after adjustment on confounding variables. Nevertheless, we have to mention that, at that period of time, patients in non‐IC group, have received only HMA. Actually, AML including t‐AML patients who are not eligible for IC are treated by combinations of HMA or cytarabine with Bcl2 inhibitors with or without targeted therapies (FLT3 inhibitors, IDH1 and 2 inhibitors, and anti‐CD47 antibody).^19^, ^51^, ^52^ In addition, magrolimab and APR‐246 showed interesting results in TP53 mutant t‐MN.^51^, ^53^ In this real‐life study, we integrated BSC patients, frequent in this category of AML, who should be included in future AML studies.

In Allo‐HCT for t‐AML setting, a positive impact of CPX‐351 on OS and TRM was observed.^21^, ^54^ Considering Allo‐HCT, we showed in our study a usual GVHD incidence but a high balanced mortality rate related to either toxicity or relapse. A comparative study between sAML/t‐AML and de novo AML undergoing Allo‐HCT showed after adjustment for ELN risk and pre‐HCT MRD status that disease subtype did not impact outcome.^55^

More recently, Baranwal et al had identified a very high‐risk score (TP53/BCOR/IDH1/ GATA2/BCORL1) for Allo‐HCT outcome with survival of 0% versus 64.6% (*p* = 0.001).^56^

Our results do not suggest t‐AML as an independent poor prognostic factor, because of the similarity of OS between t‐AML and de novo AML. This is related to a matching of IC‐treated patients on age, cytogenetics, and ELN2010 in the pair‐matched analysis of t‐AML and de novo AML. These results are similar to those obtained by Chen et al. who also performed a pair‐matched analysis between de novo AML and t‐AML after breast cancer.

This OS similarity is controversial in other studies as German–Austrian AMLSG registry study^18^ and PETHEMA registry study^57^ who did not perform a pair‐matched analysis between t‐AML and de novo AML and included all consecutive newly diagnosed AML. These two studies showed difference of characteristics between the two populations with older patients and more frequent abnormal cytogenetics in t‐AML than in de novo AML. The only similarities between all these studies is that t‐AML patients had less frequently NPM1 mutations and FLT3‐ITD. Due to pair‐matched analysis results observed, we maintain that t‐AML seem not an independent risk factor of poor outcome in younger patients who received IC.

Our study has some limitations related to its retrospective design, relatively small number of patients studied, and NGS nonavailability. Focusing on pair‐matched analysis, our results were controversial with some literature data, suggesting that t‐AML is not an independent risk factor for poor prognosis but rather an accumulation of patients whose probability of harboring other risk factors is higher. The recent ELN2022 update of AML providing an interesting hierarchical classification should allow to better characterize t‐AML and prove if this entity is an independent risk factor for poor prognosis or not.

In conclusion, the best therapeutic strategy for patients with t‐AML needs to take into consideration age, performance status, prognostic factors while integrating newly available treatment. For older t‐AML patients and young patients with comorbidities not eligible for IC and/or Allo‐HCT, HMA or cytarabine associated to venetoclax with or without targeted therapies (FLT3, IDH inhibitors, or anti‐CD47 antibody especially for TP53 mutated patients) seems to be the best therapeutic option. Considering patients eligible for Allo‐HCT, to decrease toxicity and improve results after transplantation, prior CPX‐351 based chemotherapy should be privileged. Finally, the very poor results of the BSC strategy in t‐AML and availability of new therapeutic approaches, lead us to ensure an exhaustive analysis of noneligibility criteria of t‐AML patients before allocating them to BSC strategy.

## AUTHOR CONTRIBUTIONS

**Amine Belhabri:** Conceptualization (lead); data curation (lead); formal analysis (equal); investigation (equal); methodology (equal); resources (equal); supervision (lead); validation (lead); writing – original draft (lead); writing – review and editing (lead). **MAEL HEIBLIG:** Conceptualization (equal); data curation (equal); formal analysis (equal); methodology (equal); resources (equal); validation (equal); writing – original draft (supporting); writing – review and editing (supporting). **Stephane MORISSET:** Formal analysis (lead); methodology (equal); software (lead); validation (equal); writing – original draft (equal); writing – review and editing (equal). **LILIANA VILA:** Conceptualization (supporting); data curation (supporting); resources (supporting). **CLEMENCE SANTANA:** Resources (supporting); writing – original draft (supporting). **Emmanuelle Nicolas‐Virelizier:** Resources (supporting); writing – original draft (supporting). **Sandrine Hayette:** Conceptualization (equal); data curation (equal); resources (equal). **Isabelle Tigaud:** Conceptualization (equal); data curation (equal); resources (equal). **Adriana Plesa:** Conceptualization (equal); data curation (equal); resources (equal). **Hélène Labussiere‐Wallet:** Resources (supporting); writing – original draft (supporting). **Mohamad Sobh:** Formal analysis (supporting); methodology (supporting); supervision (equal); validation (supporting); writing – original draft (supporting). **Anne‐Sophie Michallet:** Resources (supporting); writing – original draft (supporting). **Balsat Marie:** Resources (supporting); writing – original draft (supporting). **Franck Emmanuel Nicolini:** Resources (equal); writing – original draft (equal). **Yann Guillermin:** Resources (supporting); writing – original draft (supporting). **Fossard Gaëlle:** Resources (supporting); writing – original draft (supporting). **Laure Lebras:** Resources (supporting); writing – original draft (supporting). **Philippe Rey:** Resources (supporting); writing – original draft (supporting). **Lucie Jauffret‐Bertholon:** Resources (supporting); writing – original draft (supporting). **MARIE‐CHARLOTTE LAUDE:** Resources (supporting); writing – original draft (supporting). **Loron Sandrine:** Resources (supporting); writing – original draft (supporting). **Mauricette Michallet:** Conceptualization (lead); data curation (lead); formal analysis (equal); methodology (equal); resources (equal); validation (lead); writing – original draft (lead); writing – review and editing (lead).

## FUNDING INFORMATION

This study was not financially supported.

## CONFLICT OF INTEREST STATEMENT

The authors do not declare any conflict of interest.

## ETHICS STATEMENT

This study is exempt from ethical approval according to our regional IRB stating. Registry number assigned to the treatment: R201‐004‐292.

All patients who did not die did not refuse the use of their health data during their lifetime after an individual call by the physician in charge of the study (Leon Berard cancer center, Lyon) and his counterpart at Univesity Hospital Lyon Sud.

## Supporting information


Figures S1–S2

Tables S1–S2

## Data Availability

Not applicable.
