# Supplementary figures and images for: Clinical outcome of therapy‐related acute myeloid leukemia patients. Real‐life experience in a University Hospital and a Cancer Center in France
Source: Cancer Med. 2023 Aug 7;12(16):16929–44. doi: 10.1002/cam4.6322 (PMC10501294; doi:10.1002/cam4.6322)

**Figure 5**

**Supplementary figures**

**A**


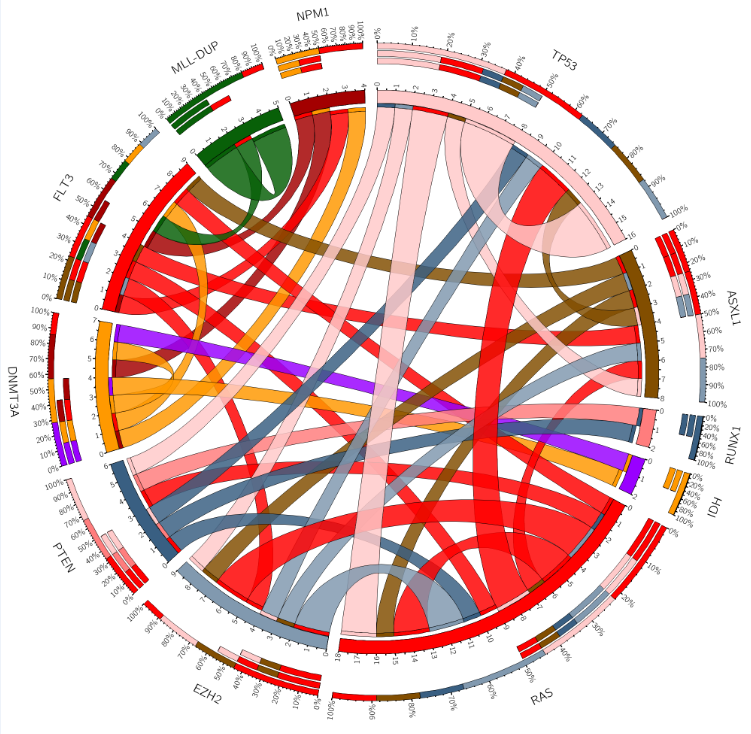


**B**


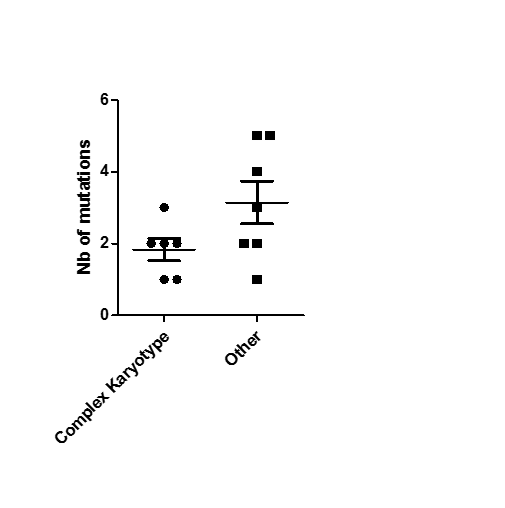


**Supplementary Figures 1A and 1B**

**
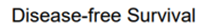

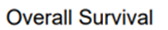
 A1 B1**

**
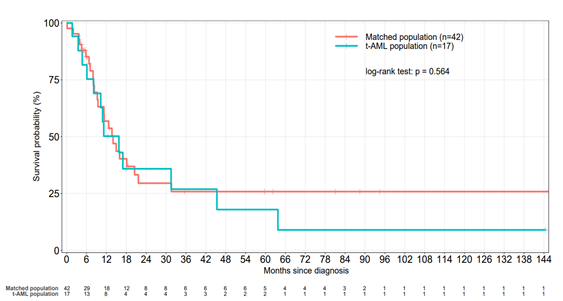
**


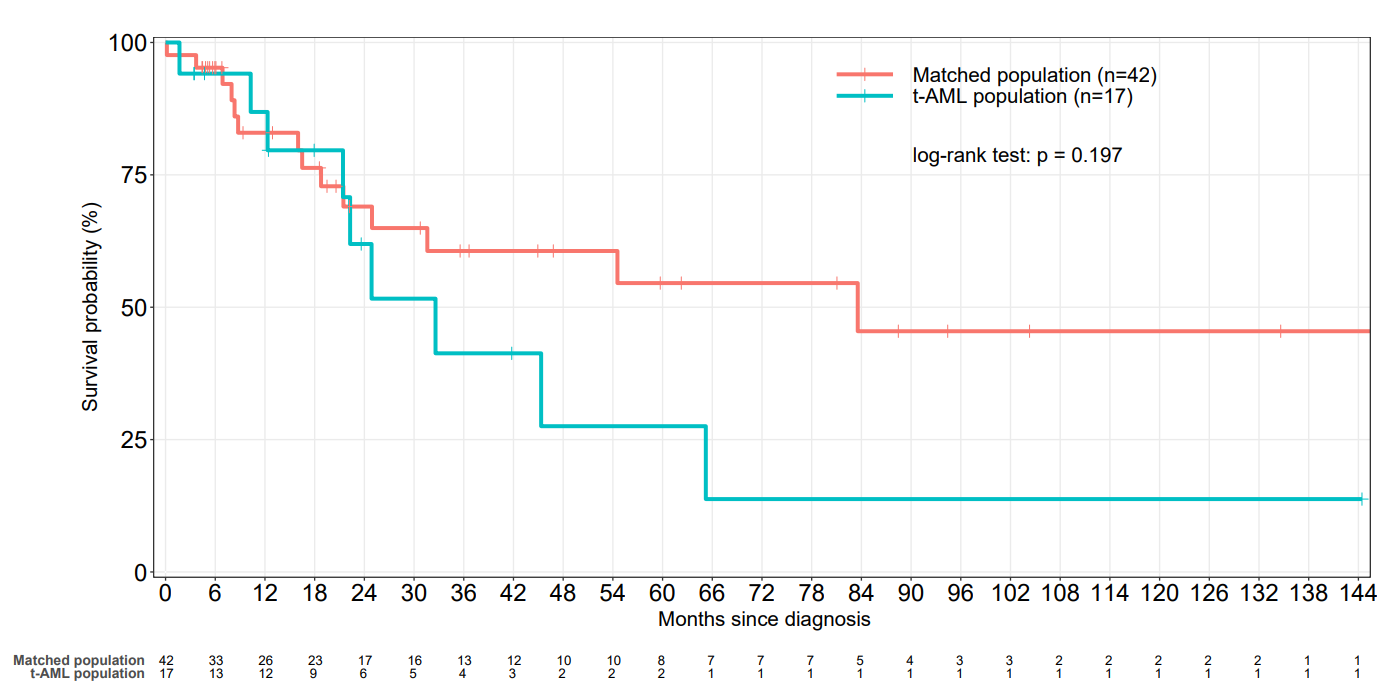


**B2**

**
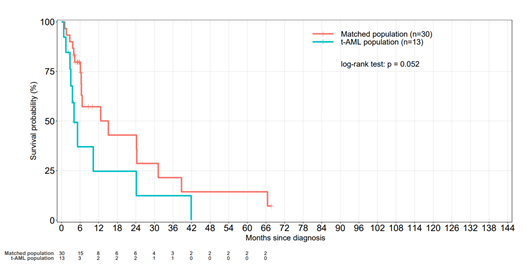
 A2**


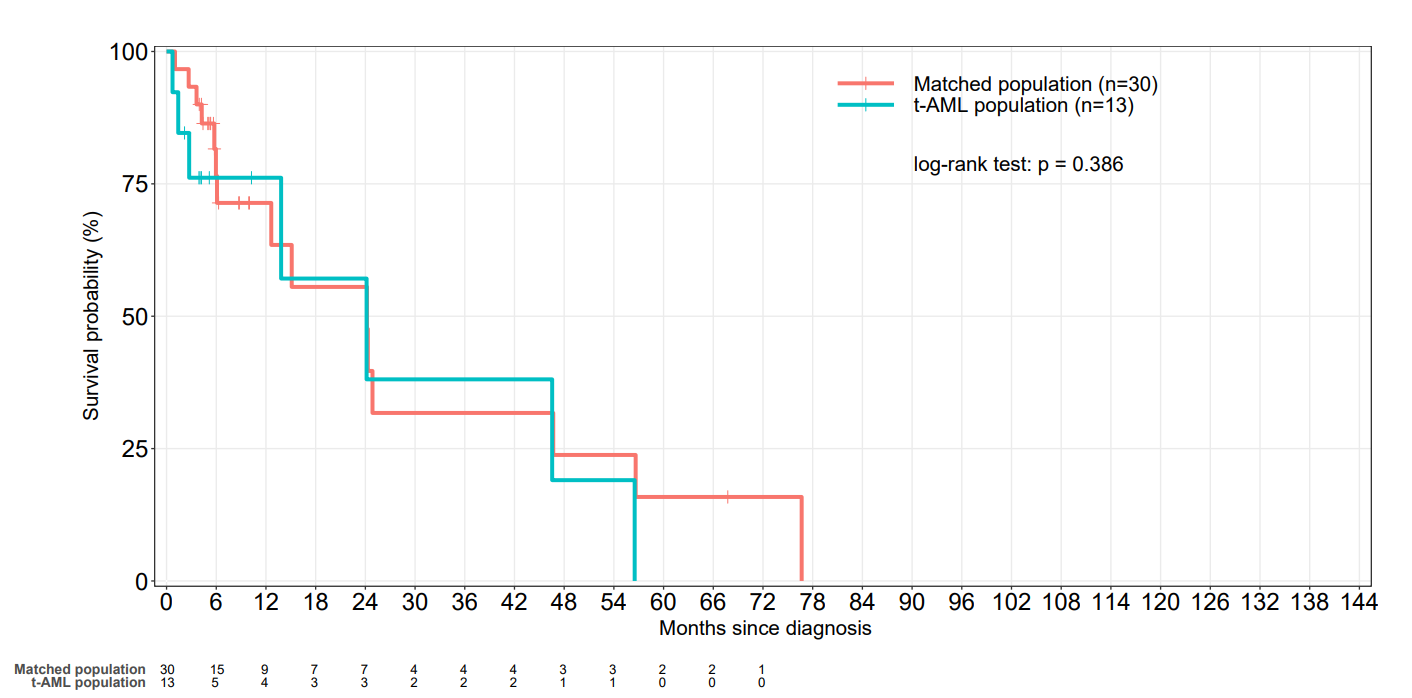


**B3**

**
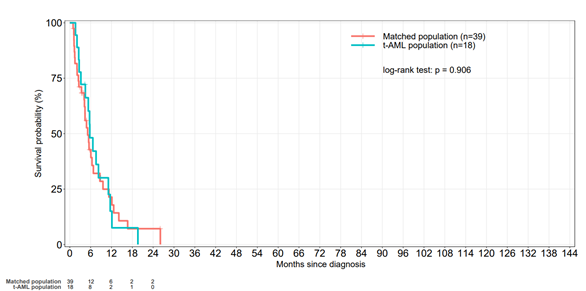
 A3**


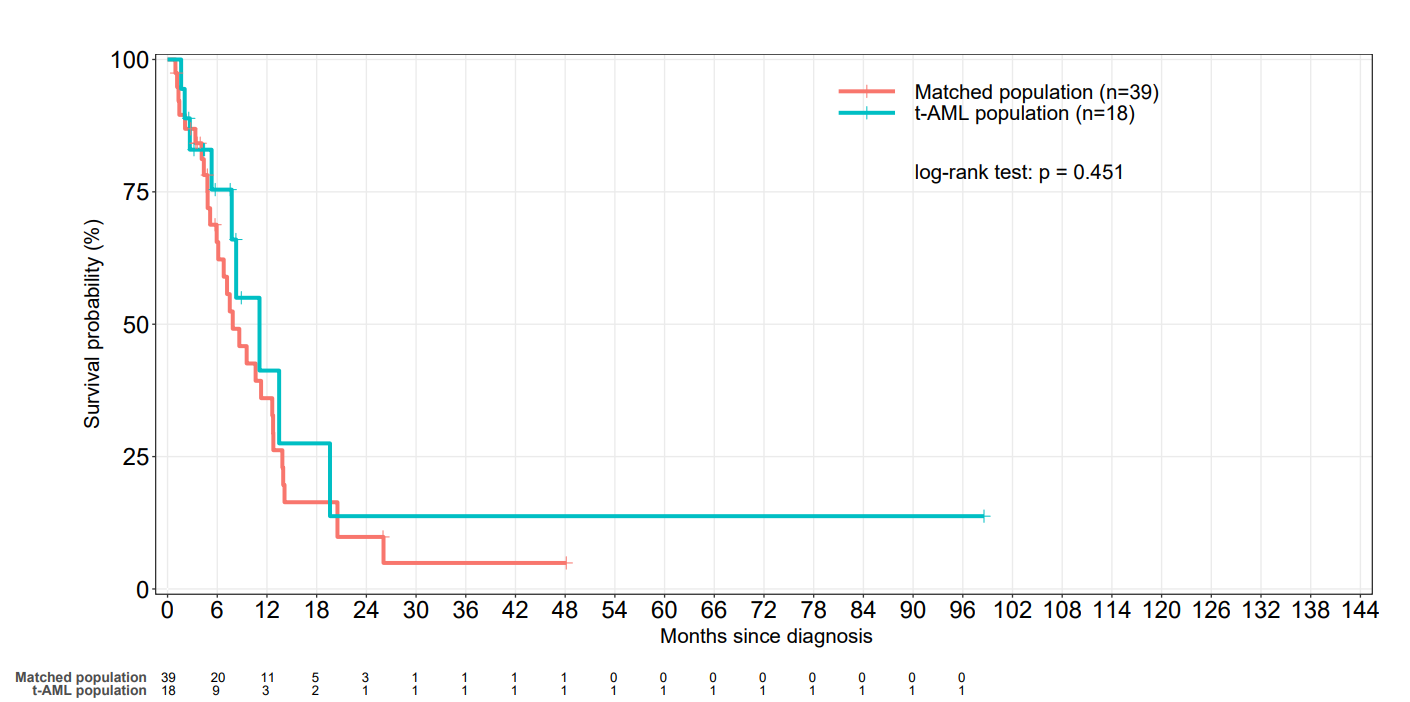


**Supplementary Figure 2**

Supplement: Supplementary file 1 — Figures S1–S2 [file CAM4-12-16929-s002.docx]
