# Supplementary material for: Clinical outcome of therapy‐related acute myeloid leukemia patients. Real‐life experience in a University Hospital and a Cancer Center in France
Source: Cancer Med. 2023 Aug 7;12(16):16929–44. doi: 10.1002/cam4.6322 (PMC10501294; doi:10.1002/cam4.6322)
Supplement: Supplementary file 2 — Tables S1–S2 [file CAM4-12-16929-s001.docx]

|  |  |  |  | *Univariate* | | *Multivariate* | |
| --- | --- | --- | --- | --- | --- | --- | --- |
| **Variable** | **Available data** | **Reference** | **Modality** | **HR [95% CI]** | **p-value** | **HR [95% CI]** | **p-value** |
| Age at diagnosis | 100 % | Continuous variable |  | 1.00 [0.98-1.02] | 0.932 | 1.00 [0.97-1.04] | 0.890 |
| Number of previous cancers | 100 % | 1 | 2 | 1.87 [0.74-4.72] | 0.183 | 1.13 [0.34-3.80] | 0.838 |
| % blasts in marrow | 92.05 % | Continuous variable |  | 1.00 [0.99-1.01] | 0.936 | 1.00 [0.99-1.02] | 0.577 |
| 2010 ELN | 90.91 % | favorable | intermediate-1 | 1.12 [0.37-3.36] | 0.837 | 1.24 [0.39-3.98] | 0.718 |
|  |  |  | intermediate-2 | 2.02 [0.69-5.96] | 0.202 | 2.57 [0.78-8.48] | 0.121 |
|  |  |  | unfavorable | 2.21 [0.81-6.08] | 0.123 | 2.20 [0.76-6.32] | 0.144 |
|  |  |  | ND | 2.45 [0.81-7.42] | 0.113 | 1.81 [0.51-6.39] | 0.358 |
| t-AML treatment | 100 % | IC | Non-IC | 1.86 [1.09-3.20] | 0.024 | 1.76 [0.77-4.05] | 0.181 |

**Extended Data Table 1: Univariate and multivariate analyses focused on confounding variables between IC and non-IC groups**

|  | **t-AML (N=55)** | **Matched cohort (N=117)** | **p-value** |
| --- | --- | --- | --- |
| Age, median (range) | 57.3 (19.1-75.3) | 63.3 (23.2-68.5) | 0.078 |
| Ratio M/F | 23/22 | 60/57 | 0.47 |
| ELN 2010 risk group, n (%)   - Favorable - Intermediate-1 - Intermediate-2 - Unfavorable - Missing | 6/55 (10.9)  12/55 (21.8)  14/55 (25.4)  19/55 (34.5)  4/55 (7.2) | 13/117 (11.1)  29/117 (24.8)  30/117 (25.6)  39/117 (33.3)  6:117 (5.4) | 0.55 |
| NPM1 mut, n (%) | 6/51 (11.7) | 19/113 (14.1) | 0.37 |
| FLT3-ITD, n (%) | 4/50 (8) | 16/113 (14.1) | 0.26 |
| HSCT, n (%) | 14/55 (27.7) | 24/117 (20.5) | 0.46 |

**Extended Data Table 2: Characteristics of patients in pair-matched analysis: t-AML versus de novo AML**
